# Differential kinetic profiles and metabolism of primaquine enantiomers by human hepatocytes

**DOI:** 10.1186/s12936-016-1270-1

**Published:** 2016-04-19

**Authors:** Pius S. Fasinu, Bharathi Avula, Babu L. Tekwani, N. P. Dhammika Nanayakkara, Yan-Hong Wang, H. M. T. Bandara Herath, James D. McChesney, Gregory A. Reichard, Sean R. Marcsisin, Mahmoud A. Elsohly, Shabana I. Khan, Ikhlas A. Khan, Larry A. Walker

**Affiliations:** The National Center for Natural Products Research, School of Pharmacy, The University of Mississippi, University, MS 38677 USA; Department of BioMolecular Sciences School of Pharmacy, The University of Mississippi, University, MS 38677 USA; Departments of Pharmaceutical Sciences and Drug Delivery, School of Pharmacy, The University of Mississippi, University, MS 38677 USA; ElSohly Laboratories, Inc., 5 Industrial Park Dr, Oxford, MS 38655 USA; Ironstone Separations, Inc., Etta, Oxford, MS 38627 USA; Military Malaria Research Program, Experimental Therapeutics Branch, Walter Reed Army Institute of Research, 503 Robert Grant Ave, Silver Spring, MD 20910 USA

**Keywords:** Enantioselectivity, Hepatocytes, Malaria, Metabolism, Primaquine enantiomers, Primaquine metabolites

## Abstract

**Background:**

The clinical utility of primaquine (PQ), used as a racemic mixture of two enantiomers, is limited due to metabolism-linked hemolytic toxicity in individuals with genetic deficiency in glucose-6-phosphate dehydrogenase. The current study investigated differential metabolism of PQ enantiomers in light of the suggestions that toxicity and efficacy might be largely enantioselective.

**Methods:**

Stable isotope ^13^C-labelled primaquine and its two enantiomers (+)-PQ, (−)-PQ were separately incubated with cryopreserved human hepatocytes. Time-tracked substrate depletion and metabolite production were monitored via UHPLC–MS/MS.

**Results:**

The initial half-life of 217 and 65 min; elimination rate constants (λ) of 0.19 and 0.64 h^−1^; intrinsic clearance (Cl_int_) of 2.55 and 8.49 (µL/min)/million cells, which when up-scaled yielded Cl_int_ of 6.49 and 21.6 (mL/min)/kg body mass was obtained respectively for (+)- and (−)-PQ. The extrapolation of in vitro intrinsic clearance to in vivo human hepatic blood clearance, performed using the well-stirred liver model, showed that the rate of hepatic clearance of (+)-PQ was only 45 % that of (−)-PQ. Two major primary routes of metabolism were observed—oxidative deamination of the terminal amine and hydroxylations on the quinoline moiety of PQ. The major deaminated metabolite, carboxyprimaquine (CPQ) was preferentially generated from the (−)-PQ. Other deaminated metabolites including PQ terminal alcohol (*m/z* 261), a cyclized side chain derivative from the aldehyde (*m/z* 241), cyclized carboxylic acid derivative (*m/z* 257), a quinone-imine product of hydroxylated CPQ (*m/z* 289), CPQ glucuronide (*m/z* 451) and the glucuronide of PQ alcohol (*m/z* 437) were all preferentially generated from the (−)-PQ. The major quinoline oxidation product (*m/z* 274) was preferentially generated from (+)-PQ. In addition to the products of the two metabolic pathways, two other major metabolites were observed: a prominent glycosylated conjugate of PQ on the terminal amine (*m/z* 422), peaking by 30 min and preferentially generated by (+)-PQ; and the carbamoyl glucuronide of PQ (*m/z* 480) exclusively generated from (+)-PQ.

**Conclusion:**

Metabolism of PQ showed enantioselectivity. These findings may provide important information in establishing clinical differences in PQ enantiomers.

## Background

The sustained utility and continued interest in primaquine (PQ), even more than six decades after its introduction into clinical medicine, underscores its unique therapeutic indications [1]. As a prototype 8-aminoquinoline, PQ is the only licensed option to treat the relapsing liver stages (hypnozoites) of *Plasmodium vivax*, in addition to its use in the prophylaxis of all forms of human malaria [2–5]. PQ is also active against mature, infective *Plasmodium falciparum* gametocytes, and there is thus current high interest in its use as a gametocytocidal drug for blocking transmission of *P. falciparum*. In areas of emerging drug resistance, primaquine has shown effectiveness against and in the prevention of the spread of artemisinin*-*resistant *P. falciparum* strains [6]. The major drawback in the clinical utility of primaquine however, is its haemolytic toxicity in individuals with genetic deficiency in glucose-6-phosphate dehydrogenase (G6PD) [4, 5].

Many attempts have been made to improve the therapeutic index of PQ and other 8-aminoquinolines [2]. PQ is a chiral drug currently used as a racemic mixture, approximating a 50:50 ratio of (+)-(*S*)- and (−)-(*R*)-enantiomers. It has been suggested that the stereo-configuration of PQ side chain may dramatically impact its metabolism, toxicity, and anti-malarial activity. More than three decades ago, Schmidt et al. suggested the exploration of the differential anti-malarial efficacy and toxicity profiles of the individual PQ enantiomers for possible improvement of the therapeutic index of PQ [7]. However, the lack of an economical stereospecific method to prepare individual enantiomers or an effective practical procedure to resolve the racemate into enantiomers delayed further studies on individual PQ enantiomers.

An efficient method for the resolution of racemic PQ mixture into individual enantiomers and findings on enantioselective activity and toxicity profiles in several animal models were recently reported [8, 9]. From these studies, it was observed that (+)-(PQ) was more active than (−)-PQ in suppressive and prophylactic anti-malarial assays as well as *Pneumocystis carinii* pneumonia efficacy assay in mice [8]. However, for radical curative assay in monkeys, previous studies have shown that both PQ enantiomers were equally effective [7, 9]. (+)-PQ caused higher acute toxicity than (−)-PQ in beagle dogs and mouse models. In monkeys, reversible liver damage was observed at higher than therapeutic (−)-PQ doses. In three different animal models (beagle dogs, humanized mouse model and monkeys) (+)-PQ caused higher methaemoglobinemia than (−)-PQ. The observed disparity of activity and toxicity profiles of PQ enantiomers in different animal models could be attributed to their differential pharmacokinetics and metabolism. Based on these results, it was suggested that (−)-(*R*)-PQ enantiomer may have a better safety margin than the currently used racemate and proposed a “phase I” type human study to compare pharmacokinetics and tolerability study of PQ enantiomers [7, 9].

It had long been speculated that PQ needed metabolic activation due to its lack of in vitro activity and toxicity, and the observed delayed onset of action in animal models. PQ treatment failures were reported in subjects with impaired cytochrome P_450_ 2D6 (CYP2D6) function [10]. Subsequent studies using a CYP2D knock-out mouse model found evidence that CYP2D metabolism was a prerequisite for the causal prophylactic activity of PQ [11]. Differential metabolism of PQ enantiomers in vitro by recombinant human CYP2D6 was previously reported [12]. The principal CYP2D6-dependent metabolic pathway of PQ was found to involve the hydroxylation of the quinoline ring yielding 2-, 3-, and 4-hydroxyprimaquine, PQ-5,6-orthoquinone and dihydroxyprimaquine as the major metabolites. PQ-5,6-orthoquinone appeared to be the oxidative degradation product of 5-OH-PQ. Synthetic 5-OH-PQ spontaneously undergoes rapid oxidation sequentially to PQ-5,8-quinone-imine and PQ-5,6-orthoquinone [13]. Both quinone-imine and orthoquinone can generate reactive oxygen species (ROS) through redox cycling, hence are possible candidates as the active metabolites of PQ. ROS have shown to be responsible for both antiparasitic activity and haemotoxity. The rate and extent of the generation of these metabolites differ significantly with individual PQ enantiomers [12]. Primaquine-5,6-orthoquinone was twice as abundant from CYP2D6-catalyzed metabolism of (+)-PQ metabolism compared to (−)-PQ. The rate of metabolism in vitro with recombinant human CYP2D6 was significantly higher for (+)-PQ compared to (−)-PQ.

In addition to the CYP-mediated oxidation of the quinoline ring, oxidative deamination of the terminal amine by monoamine oxidase A (MAO) has been identified as the other major metabolic pathway of PQ. Oxidation of PQ by MAO results in formation of an aldehyde metabolite, which is further oxidized or reduced to the stable metabolites namely, carboxyprimaqine (CPQ) and PQ-alcohol, respectively. CPQ had been identified as the major circulating human metabolite of racemic PQ [14, 15]. Pharmacokinetic studies of racemic PQ in mice, monkeys and humans have shown that CPQ rapidly appeared in the plasma and was predominantly the (−)-form [9, 15, 16]. The current study is aimed at characterizing the kinetics of metabolic profiles of the individual PQ enantiomers in human hepatocytes, where both the CYP and MAO pathways are present.

The primary aim of this study was therefore to characterize the metabolism of the individual enantiomers of PQ in human hepatocytes, while profiling the enantioselective metabolites generated. This is expected to provide projections of enantioselective metabolic profiles in human. To facilitate identification of metabolites a 1:1 mixture ^13^C-(C6)-labelled and normal ^12^C-PQ was used in this study.

## Methods

HPLC-grade acetonitrile and methanol were purchased from Fisher Scientific (Fair Lawn, NJ, USA). Water for the HPLC mobile phase was purified using a Milipore Synergy Water Purification System (Milipore SAS, Molsheim, France). PQ diphosphate and formic acid were purchased from Sigma (St Louis, MO, USA).

### Synthetic chemicals

^13^C(6)-labelled PQ was prepared [17] and resolved into (+)-(*S*)- and (−)-(*R*)- enantiomers as previously reported [8]. CPQ [18] and CPQ-lactam [19] were prepared using the procedures reported. Synthesis of 2-, 3-, and 4-OHPQ and PQ alcohol were reported earlier [12].

### Synthesis of primaquine alcohol glucuronide

#### Primaquine alcohol-β-d-2,3,4-O-triacetylglucuronide methyl ester

Ag_2_CO_3_ (414 mg, 1.5 mM) was added potion wise for 2 h to a stirring mixture of acetobromo-α-d-glucuronic acid methyl ester (398 mg, 1 mM), and primaquine alcohol (390 mg, 1.5 mM) in dry toluene (10 mL) at 75 °C and the reaction was left overnight. The reaction mixture filtered through Celite, and the filtrate was evaporated to dryness The product was purified by column chromatography on silica gel with hexane:EtOAc (90:10) to give primaquine alcohol-O-β-d-2,3,4-O-triacetylglucuronide methyl ester (410 mg).

^1^H NMR δ (CDCl3): 1.08 (3H, d, J = 4.0 Hz), 1.49 (4H, m), 1.82 (3H,s), 1.84 (6H, s), 3.41 (4H, m), 3.53 (3H, s), 3.66 (3H, s), 4.27 (1H, d, J = 8.0 Hz), 4.95 (1H, d, J = 8.0 Hz), 5.05 (1H, dd, J = 6.0, 8.0 Hz), 5.35 (1H, dd, J = 6.0. 8.0 Hz), 6.10 (1H,s), 6.13 (1H, s), 6.21 (1H, d, J = 4.0 Hz), 7.07 (1H, dd, J = 6.0, 4.0 Hz), 7.70 (1H, d, J = 6.0 Hz), 8.29 (1H, d, J = 4.0 Hz); HRESIMS [M + H]^+^*m/z* 577.2423 (calculated for (C_28_H_36_N_2_O_11_ + H)^+^ 577.2397).

#### Primaquine alcohol-O-β-d-glucuronide

Primaquine alcohol-O-β-d-2,3,4-O-triacetylglucuronide methyl ester (350 mg) was dissolved in a mixture of MeOH/H2O (5:1)(10 mL) stirred at 0 °C, added ethyl di-isopropyl amine (2 mL) and continued stirring overnight at room temperature. Solvent was evaporated under vacuum and the residue was chromatographed on reverse phase silica (C-18) column with H_2_O:MeOH to give primaquine alcohol-O-β-d-glucuronic acid.

^1^H NMR δ (CD_3_OD): 8.67 (1H, brs), 8.44(1H, d, J = 6.0 Hz), 7.64 (1H, m), 6.48 (1H, s), 5.14 (1H, s), 4.52 (1H, d, J = 8.0 Hz), 3.88 (3H, s), 3.84 (1H, m), 3.68 (1H, m), 3.40 (1H, m), 3.34 (1H, m), 3.20 (2H,m), 1.80 (4H,m), 1.30 (3H, d, J = 6.0 Hz); HRESIMS [M + H]^+^*m/z* 437.1884 (calculated for (C_21_H_28_N_2_O_8_ + H)^+^ 437.1924).

### Synthesis of carboxyprimaquine-β-d-glucuronide

#### Benzyl 1-O-levulinyl-d-glucuronate

*N-*Methyl mopholine (0.25 mL) was added to a mixture of benzyl d-glucuronate (426 mg, 1.5 mM), levulinic acid (185 mg, 1.6 mM) and HATU (380 mg, 1 mM) in anhydrous acetonitrile (10 mL) and the reaction mixture was stirred 2 h at ambient temperature. Reaction was quenched with Ambalite H^+^ resin, filtered and the residue was thoroughly washed with CH_2_Cl_2_. Combined organic extract was evaporated to dryness and chromtographed on silica column with CH_2_Cl_2_:MeOH (96:4) to give benzyl 1-O-levulinyl-d-glucuronate (550 mg).

^1^H NMR δ (CDCl_3_): 2.06 (3H, s), 2.72-2.48 (4H, m), 3.80-3.58 (4H, m), 4.02 (1H, d, J = 9.6 Hz), 5.15 (2H, s), 5.60 (1H,d, J = 7.6 Hz), 7.30 (5H, m).

#### Carboxyprimaquine-β-d-glucuronide benzyl ester

NaBH_4_ was added portion wise to a mixture of benzyl 1-O-levulinyl-d-glucuronate (275 mg, 0.72 mM) and 6-methoxy-8-aminoquinoline (128 mg, 0.8 mM) in glacial acetic acid (5 ml) at 20 °C under stirring while monitoring the reaction by TLC. Upon completion, the reaction was quenched with ice/water, neutralized with 10 % NaOH and extracted with CH_2_Cl_2_. The organic extract was dried on anhydrous Na_2_SO_4_, evaporated to dryness. The product was purified by chromatography on silica gel and elution with CH_2_Cl_2_/MeOH (5 %) to give carboxyprimaquine-β-d-glucuronide benzyl ester (260 mg).

#### Carboxyprimaquine-β-d-glucuronide

Carboxyprimaquine-β-d-glucuronide benzyl ester (210 mg) and 10 % Pd/C in EtOH (10 mL) was hydrogenated with H_2_ (20 psi) for 2 h. Reaction mixture was filtered through Celite purified by silica column chromatography with CH_2_Cl_2_:MeOH to give carboxyprimaquine-β-d-glucuronide (140 mg).

^1^H NMR δ (CD_3_OD): 8.48 (1H, brs), 8.02 (1H, d, J = 8.0 Hz), 7.35 (1H, m), 6.38 (1H, d, J = 7.6 Hz), 5.51 (1H, m), 3.91 (1H, m), 3.84 (3H,s), 3.68 (1H, m), 3.46 (1H, m), 3.39 (1H, m), 3.30 (1H, m), 2.54 (2H, m), 1.90 (2H, m), 1.25 (3H, d, J = 6.4 Hz): ^13^C NMR δ (CD_3_OD): 19.1, 30.2, 30.8, 54.3, 71.6, 72.1, 75.5, 76.0, 94.0, 97.4, 121.5, 130.1, 135.1, 143.8, 144.5, 159.5, 172.4: HRESIMS [M + H]^+^*m/z* 451.1776 (calculated for (C_21_H_26_N_2_O_9_ + H)^+^ 451.1717).

### Synthesis of (S)-primaquine-*N*-carbamoyl-β-d-glucuronide

#### (S)-primaquine-*N-*carbamoyl-2,3,4-O-triacetyl-β-d-glucuronide methyl ester

A solution of 4-dimethylamino pyridine (112 mg, 0.92 mM) in anhydrous CH_3_CN (5 mL) was added dropwise to a solution of di-*tert*-butyl dicarbonate (1.2 g, 5.5 mM) in anhydrous CH_3_CN (5 mL) at 0 °C under stirring. After 10 min, a solution of (+)-(*S*)-primaquine free amine (1.2 g, 4.6 mM) in anhydrous CH_3_CN (5 mL) was added dropwise and the reaction mixture was stirred for further 30 min at 0 °C. Solvent was evaporated under reduced pressure and the resulting dark yellow oily residue was dissolved in toluene (5 mL), cooled to 0 °C, treated dropwise with a solution of methyl 2,3,4-tri-O-acetyl-d-glucuronnte (1.8 g, 5.5 mM) in toluene (5 mL) followed by triethylamine (1.2 mL) and stirred for 2 h at 0 °C and another 2 h at room temperature. Solvent was evaporated under reduced pressure and the dark brown residue was chromatographed on silica column and eluted with ethyl acetate/hexane (40:60) to give (S)-primaquine-*N*-carbamoyl -2,3,4-O-triacetyl-β-d-glucuronide methyl ester (1.80 g).

^1^H NMR δ (CDCl_3_): 1.25 (3H, d, J = 6.8 Hz), 1.60 (4H, m), 1.99 (9H, brs), 3.15 (2H, m), 3.58 (1H, m), 3.66 (3H, s), 3.85(3H, s), 4.12 (1H, d, J = 10, Hz), 5.31-5.09 (4H, m), 5.67 (1H, d, J = 8.4 Hz), 5.94 (1H, brd, J = 8.4 Hz), 6.23 (1H, s), 6.30 (1H, s), 7.27 (1H, dd, J = 8.4, 7.6 Hz), 7.88 (1H, d, J = 8.4 Hz), 8.49 (1H, d, J = 4.4 Hz); HRESIMS [M + H]^+^*m/z* 620.2451 (calculated for (C_29_H_37_N_3_O_12_ + H)^+^ 620.2455).

#### (S)-primaquine-*N-*carbamoyl-β-d-glucuronide

Ethyldiisopropyl amine (6 mL) was added to a solution of primaquine-*N*-carbamoyl-2,3,4-O-triacetyl-β-d-glucuronide methyl ester (700 mg, 1.12 mM) in MeOH (30 mL) and H_2_O (6 mL) at 0 °C while stirring. After 10 min at 0 °C, cooling bath was removed and stirring was continued overnight. Solvent was removed under reduced pressure and the residue was dissolved in MeOH (2 mL), introduced to a Sephadex column (4 × 30 cm) and eluted with methanol (0.5 mL/min) to give (S)-primaquine-*N*-carbamoyl-β-d-glucuronide (284 mg) as a pale yellow powder.

^1^H NMR δ (D_6_-DMSO): 1.18 (3H, d, J = 6.4 Hz), 1.60 (4H, m), 3.33-2.99 (7H, m), 3.66 (3H, s), 3.59 (2H, m), 3.80 (3H, s), 5.16 (1H, d, J = 8.4 Hz), 6.11 (1H, d, J = 8.4 Hz), 6.24 (1H, d, J = 2.0 Hz), 6.44 (1H, d, J = 2.0 Hz), 7.40 (1H, dd, J = 8.0, 8.0 Hz), 8.05 (1H, d, J = 8.4 Hz), 8.51 (1H, d, J = 3.6 Hz); ^13^C NMR δ (D_6_-DMSO): 20.6, 26.6, 33.8, 47.5, 55.4, 72.3, 72.8, 74.8, 77.1, 92.1, 95.4, 96.6, 122.6, 130.0, 135.0, 135.3, 145.1, 144.7, 155.6, 159.4, 173.1; HRESIMS [M + H]^+^*m/z* 480.1954 (calculated for (C_22_H_29_N_3_O_9_ + H)^+^ 480.1982).

### Hepatocytes

Pooled mixed-gender cryopreserved metabolism-qualified (CYP1A2, 2B6, 2C8, 2C9, 2C19, 2D6, 3A4, UGT2B7, UDP-glucuronosyltransferase) primary human hepatocytes (catalog number 454504) sourced from free donors (who were free of HIV, HBV, HCV and other liver-related diseases) were purchased from Corning Life Sciences (Woburn MA, USA) and stored in liquid nitrogen until use.

### Hepatocytes incubations and viability measurements

The cryopreserved hepatocytes were thawed and the cells were suspended in recovery medium (Corning Life Sciences). According to the supplier’s instructions, the suspended cells were centrifuged (300*g*, 5 min), the recovery medium discarded and the cells were re-suspended in the plating medium. Viability of the suspended hepatocytes was computationally determined based on cell counts using a Bio-Rad automated cell counter (Hercules, CA, USA). The cell density was adjusted to approximately 1X10^6^ viable cells/mL in the plating media.

PQ, racemic and enantiomers, prepared as a 1:1 mixture of 12-C and 13-C-labelled compounds, were separately incubated at a final concentration of 20 µM in the cell-containing plating media using the 48-well plate at 37 °C under a humidified atmosphere of 95 % air and 5 % CO2 in an Eppendorf incubator (Hauppauge, NY, USA) attached with a shaker set at 75 rpm. A separate control incubation of PQ and the two enantiomers in the cell-free hepatocyte media was performed to detect any non*-*enzymatically generated product. Metabolic reactions were halted by the addition of two volumes of ice-cold methanol containing 0.5 µg/mL 6-d_3_-methoxyprimaquine as an internal standard at predetermined time intervals. This mixture was vortexed and kept at −20 °C for at least 4 h before further analysis. Parallel incubation of substrate-free hepatocytes was performed. At each time point, aliquots were taken for cell viability measurement.

In preparation for HPLC–MS analysis, samples were centrifuged (10,000*g*, 10 min) and clear supernatants were dried using a Speedvac. The dried samples were re-suspended in 150 µL methanol, centrifuged and clear supernatants were transferred to HPLC sample vials for analysis.

### Detection, identification and quantification of metabolites

The analytical method for simultaneous analysis of primaquine and its metabolites using the Ultra-High Performance Liquid Chromatography-Mass Spectrometry (UHPLC-QToF-MS), as earlier developed and reported was employed in this study [16]. Typically, total separation and elution of the analytes was achieved within 10 min retention time, using the Acquity UPLC™, BEH Shield RP18 column (100 mm × 2.1 mm I.D, 1.7 µm) equipped with an LC-18 guard column (Vanguard 2.1 × 5 mm, Waters Corp., Milford, MA, USA) on an ACQUITY UPLC system (Waters Corporation, Milford, MA, USA) to which a conditioned auto-sampler (at 20 °C) was attached. Details of the chromatography set-up are published [16].

Metabolites in the accurate mass data were found using the Metabolynx^®^ software. The data were searched using predicted metabolite mass, mass defects, isotope, and fragmentation patterns. Each sample was subjected to data acquisition in full scan and data-dependent positive MS/MS, targeted MS/MS (ESI positive ionization mode) and high-resolution MS (HRMS) modes using the Waters ACQUITY™ XEVO QTOF Mass Spectrometer (Waters Corporation, Manchester, UK) connected to the UHPLC system via an electrospray ionization (ESI) interface. Metabolites were distinguished from artifacts and unrelated products by the presence of twin peaks with a mass difference of six, arising from the six labels in the 1:1 mixture of 12-C and 13-C-labelled PQ. The Identification of each metabolite was assisted by its HRMS data, which were used to calculate their elemental compositions. The full scan mass data were screened and filtered using Waters MetaboLynx XS software. The qualitative metabolite identification was performed using this software package.

### *In vitro* elimination calculations

Initial half-life (t^1^/_2_) and elimination rate constants (λ = ln2/t^1^/_2_) of PQ in hepatocyte incubates were calculated by log-linear regression of PQ concentrations profiled against time using data from the sampling points of the 20 µM PQ incubates [20]. The intrinsic clearance in vitro (CLint in vitro) was calculated from λ and the cell density in the respective incubation (number of viable hepatocytes per mL at time zero) and scaled up to the intrinsic clearance in vivo (CLint in vivo) using the human liver mass (25.7 g/kg body mass) and the hepatocellularity (number of hepatocytes per gram of liver = 99 millions cells/g liver) [21]: CLint in vitro = λ/cell density. CLint in vivo = CLint in vitro × liver mass × heptocellularity. From CLint in vivo and the hepatic blood flow [Q (20.7 mL/min)/kg body mass], the hepatic metabolic blood clearance (CLh,b) was predicted using the well-stirred model: CLh,b = (CLint in vivo × Q)/(CLint in vivo + Q) [21].

Since this estimation was for comparisons between enantiomers only, no corrections were made for the free fractions in vitro or in vivo—i.e. they were assumed to be identical [21].

### Statistical analysis

Intra- and inter-day variations in the data obtained from replicated experiments were assessed for significance. Replicate agreements were within 5 % variations. All results were statistically analyzed for significance by Student’s t test using GraphPad Prism (GraphPad Software, San Diego, CA).

## Results

**Fig. 1:**
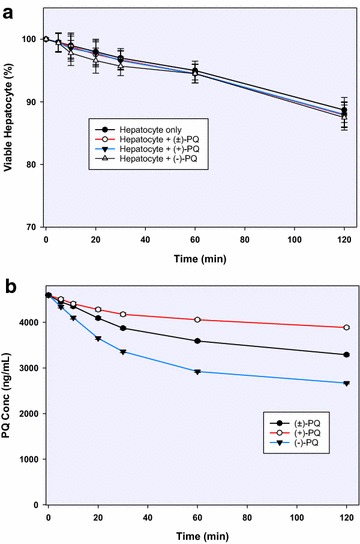
**a** Hepatocyte viability time course determined through cell counts in the presence and absence of (±)-primaquine and its (+)- and (−)-enantiomers; **b** differential depletion of the 20 µM racemic (±)-primaquine and its (+)-, and (−)-enantiomers in primary human hepatocytes (1 million cells/mL) after 2 h incubation. Each point represents values mean ±SD (n = 4)

PQ was incubated in vitro with human hepatocytes (1 million cells/mL) at a final concentration of 20 µM. Substrate concentrations, as initially titrated, showed optimal metabolism at 20 µM (Km = 37 µM). Although this is higher than expected peak plasma concentrations in humans as we earlier reported (<2 µM) [15], it approximates expected liver concentrations of PQ, which we observed to be—at therapeutic doses about 20 times higher than plasma in our studies in mice [22]. The viable cell counts in the drug-free incubation at the predetermined time-points were similar to those of the PQ-containing incubates, suggesting the absence of PQ-induced direct hepatocellular toxicity (Fig. 1a). Hepatocyte viability at the 2 h termination time was 88 % of the starting value, an indication that the integrity of the hepatocytes was not compromised through the incubation period.

### Intrinsic metabolic clearance from hepatocytes

Substrate depletion and metabolite production were monitored via UHPLC-MS/MS. By 2 h, 30, 16 and 42 % of PQ, (+)-PQ and (−)-PQ, respectively, were depleted (Fig. 1b).

The initial half-life, calculated by log-linear regression of PQ concentrations profiled against time using data from the sampling points of the 20 µM incubates, was 217 and 65 min for (+)- and (−)-PQ, with elimination rate constants (ʎ) of 0.19 and 0.64 h^−1^ respectively. The in vitro intrinsic clearance, calculated from ʎ and the cell density in the respective incubations, was 2.55 and 8.49 (µL/min)/million cells, which when upscaled in vivo based on known human hepatocellularity (hepatocytes/g liver) and liver mass (g/kg body mass) yielded 6.49 and 21.6 (mL/min)/kg body mass respectively for (+)- and (−) PQ. Extrapolation to in vivo human hepatic blood clearance was performed using the well-stirred liver model, which showed that the rate of hepatic clearance of (+)-PQ was only 30 % that of (−) PQ (Table 1).

**Table 1:** The intrinsic clearance of PQ and its enantiomers in human hepatocytes

	(±)-PQ	(+)-PQ	(−)-PQ
Time interval (min) for calculation of elimination rate constant	0–60	0–60	0–60
λ (h^−1^)^a^	0.34	0.19	0.64
t½ (h)^b^	2.03	3.62	1.09
t½ (min)	121.8	217	65
CLint in vitro [(mL/h)/million cells^c^]	0.273	0.153	0.509
CLint in vitro [(µL/min)/million cells]	4.55	2.55	8.49
CLint in vivo [(L/h)/kg body mass]^d^	0.69	0.39	1.30
CLint in vivo [(mL/min)/kg body mass]	11.58	6.49	21.60
CL_h,b_ [(L/h)/kg body mass]^e^	0.45	0.30	0.63
CL_h,b_ [(mL/min)/kg body mass]	7	5	11
CLh,b (% hepatic blood flow)^f^	36	24	51

*λ* elimination rate constant,* Clint* intrinsic clearance,* CL*_*h,b*_ hepatic blood clearance

^a^λ is taken as the -slope of the linear portion of the log-linear regression (0-60 min)

^b^Half-lives computed from ${\lambda}({\text{t1}}/2 = {\text{ln}}(2)/\lambda$

^c^ $$({\text{ln2/T1/2}})/{\text{mio viable cells}} \times 1000$$

^d^ $( {\text{CLint}} /1000) * ({\text{cells/g/liver}})* ({\text{g liver / kg body weight}})$. Hepatocellularity is given as 99 million cells/g liver; average human liver weight is 25.7 g/kg body mass

^e^ ${\text{CLh}} = ( {\text{Qh}} * {\text{CLint}},{\text{scaled}})/ ( {\text{Qh}} + {\text{CLint}},{\text{scaled}})$ *Qh* hepatic blood flow (well-stirred liver model) is given as 20.7 (mL/min)/kg body mass

^f^Qh (mL/min/kg body weight): 55 (rat); 30.9 (dog); 43.6 (monkey); 20.7 (human)

### Differential kinetics and profile of metabolites generated from PQ enantiomers

**Fig. 2:**
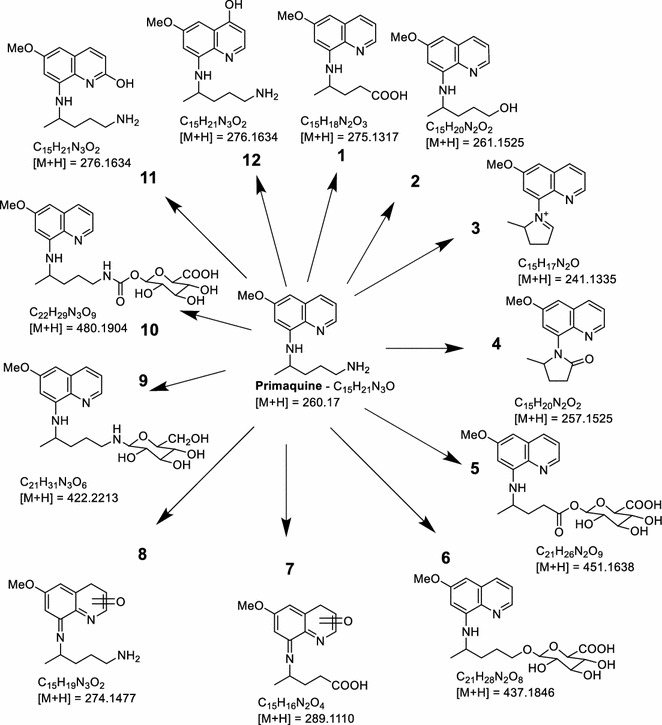
Putative identities and predicted structures of primaquine metabolites generated in human hepatocytes, as determined through MS/MS fragmentation, twin peak detection on the UHPLC chromatogram and prediction by Waters’ Metabolynx^®^ software package

**Fig. 3:**
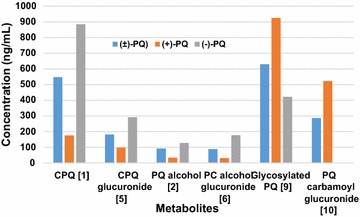
The relative concentrations of the major metabolites generated by primaquine and its enantiomers at 1 h incubation time point in primary human hepatocytes

From the metabolite profile, two major routes of metabolism were observed as earlier reported: oxidative deamination of the side chain terminal amine; and hydroxylations on the quinoline moiety [16]. The metabolites generated through both the pathways were further metabolized by phase II conjugation pathways (Fig. 2). The comparative concentrations of the metabolites generated at 1 h post-incubation is presented in Fig. 3.

**Fig. 4:**
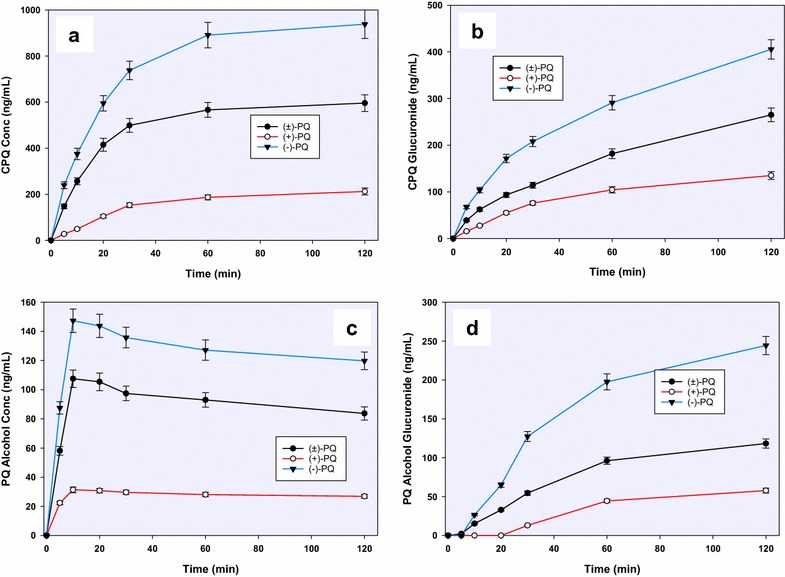
A time-course analysis of the in vitro generation of **a** carboxyprimaquine (*1*), **b** primaquine alcohol (*2*) and **c**, **d** their glucuronide conjugates (*5, 6*), from (+)-, (−)- and (±)-primaquine in human hepatocytes. Each point represents value mean ±SD of four observations

**Fig. 5:**
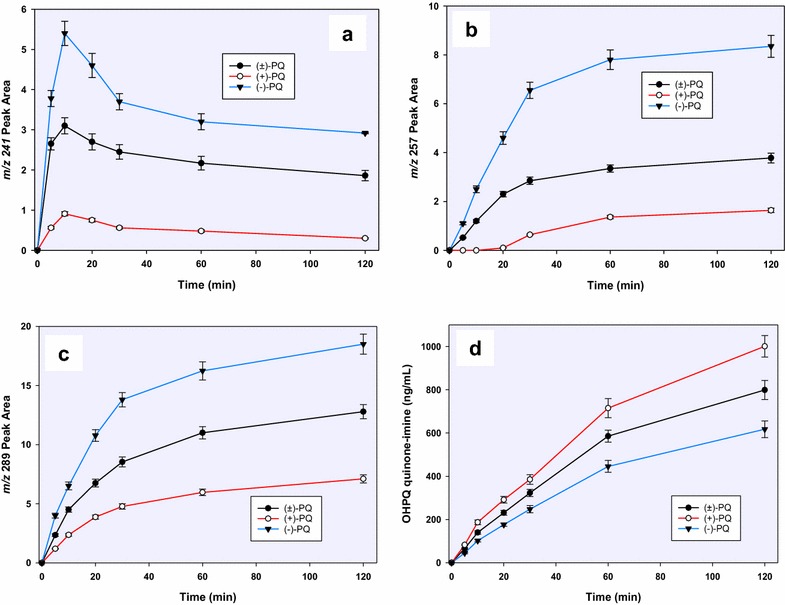
A time-course analysis of **a** cyclized PQ aldehyde (*m/z* 241) (*3*), **b** cyclized carboxyprimaquine (*m/z* 257) (*4*), **c** hydroxylated CPQ quinone-imine (*m/z* 289) (*7*) and **d** hydroxyprimaquine quinone-imine (*m/z* 274) (*8*) differentially generated from (+)-, (−)- and (±)-primaquine in vitro in human hepatocytes. Each point represents value mean ±SD of four observations

**Fig. 6:**
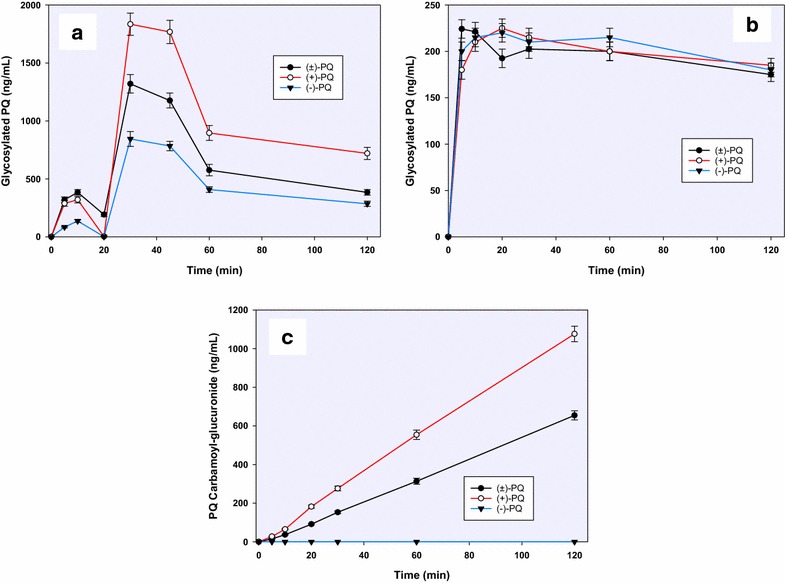
A time-course analysis of **a** glycosylated primaquine (*9*) generated through the activity of human hepatocytes; **b** non*-*enzymatic generation of primaquine-glucose conjugates observed following the incubation of primaquine and its metabolites in cell-free hepatocyte media and; **c** primaquine carbamoyl-glucuronide (*10*) differentially generated from (+)-, (−)-and (±)-primaquine in vitro in human hepatocytes. *Each point* represents value mean ±SD of four observations

The major deaminated metabolite, CPQ (Fig. 2—1) (75 % of which was generated within 30 min), was preferentially generated from the (−)-PQ, when compared to (+) PQ (about 8:1 at early time points) (Fig. 4a). This metabolite has been shown earlier to be the major product of PQ biotransformation circulating in the plasma of mice [23], monkeys [9] and humans [15]. It is formed through the oxidative deamination of the 8-*N-*aminoalkyl side chain of PQ to an intermediate PQ aldehyde which is further converted to the carboxylic acid by an aldehyde dehydrogenase. The PQ aldehyde formed from PQ may also undergo a reduction reaction to form the primaquine alcohol (*m/z* 261; Fig. 2—2), or cyclization of the side chain through the loss of a water molecule (*m/z* 241, Fig. 2—3). Further metabolism of CPQ involves the elimination of water and cyclization to the 8-amino group forming a lactam product (*m/z* 257, Fig. 2—4). CPQ and the PQ alcohol undergo conjugation reactions with glucuronides (*m/z* 451 and 437 respectively; Fig. 2—5, 6) is also formed from hydroxylated CPQ. These metabolites of the oxidative deamination of the amino side chains were preferentially generated from the (−)-PQ compared to (+)-PQ (Fig. 5a, b, c).

A major product of the quinoline ring oxidation identified was the one that corresponds to an *m/z* 274, formed by hydroxylation of PQ (Figs. 2—8) and subsequent dehydrogenation to the quinone-imine. This ring oxidation product was preferentially formed from (+)-PQ compared to (−)-PQ (2:1) (Fig. 5d). Site of hydroxylation on the quinolone ring could not be established unequivocally at present, but based on other studies, hydroxylation of the 5-position is unlikely due to instability of this hydroxylation product which is rapidly converted to PQ-5,6 orthoquinone. Three other metabolites corresponding to *m/z* 274 were also observed, which were exclusively generated by (+)-PQ.

In addition to the ring hydroxylations and side-chain terminal amine oxidative deamination pathways, two other metabolites were identified, which were generated through conjugation directly on the PQ side chain terminal amine. A prominent conjugate (*m/z* 422; Fig. 2—9), identified as a glycosylated PQ, was generated. Formation of this metabolite showed a biphasic increase, peaking by 30 min. This metabolite was preferentially generated from (+)-PQ rather than (−)-PQ (Fig. 6a). This glycosylated conjugate of PQ was also formed non*-*enzymatically in the cell-free media (Fig. 6b). A linearly accumulating metabolite (*m/z* 480; Fig. 2—10) over the 2 h time course was identified as an *N-*carbamoyl glucuronide of PQ, and was exclusively generated from (+)-PQ (Fig. 6c).

Other metabolites were detected but with too minute peak areas to profile. These include trace amounts of 2- and 4-hydroxyprimaquine (Fig. 2—11 and 12), which were exclusively generated from the (+)-PQ. Figure 2 presents the putative structural identities of the metabolites of PQ in human hepatocytes.

## Discussion

Pharmacological and toxicological activities of PQ have been attributed to the metabolites of the drug, presumably generated through CYP-mediated pathways [24, 25]. Until recently, understanding regarding the nature of metabolites and pathways involved in formation of these metabolites has been incomplete. Application of stable ^13^C isotope labeling of PQ, development of sensitive UHPLC-MS/MS methods for analysis and availability of synthetic metabolites have provided the tools necessary for more accurate identification and quantification of PQ metabolites [16]. However, the situation has been further complicated by the reports regarding differential pharmacological, toxicological and pharmacokinetics profile of enantiomers of PQ [7–9, 15]. Therefore, it is important to get a more complete picture of the metabolism and pharmacokinetics of PQ especially the differential qualitative, quantitative and kinetic profiles of the metabolites generated from individual PQ enantiomers. In the current study, the enantiomers of PQ have been shown to have differential metabolic and pharmacokinetic profiles in human hepatocytes in vitro.

The results presented herein have predicted that the initial half-life (as well as other pharmacokinetic parameters derived therefrom) of (+)-PQ was more than three times longer than that that of (−)-PQ. MAO and CYP are known to be involved in the phase I hepatic metabolism of PQ [26]. A majority of the metabolites identified in this study appeared to be generated through MAO-mediated oxidative deamination of PQ. CPQ, the product of the sequential oxidative deamination of the 8-*N*-aminoalkyl side chain by MAO and oxidation of the resulting PQ-aldehyde was the most abundant primary metabolite of PQ and was preferentially generated from the (−)-PQ (about 8 times higher than in (+)-PQ at the early time points). These results support our previous data on in vivo pharmacokinetics of PQ enantiomers in rodents, primates and humans as well in vitro results with freshly isolated human hepatocytes [9, 15]. It is noteworthy that all the metabolites formed via the oxidative deamination of the 8-*N*-aminoalkyl side chain were consistently more abundant from the (−)-PQ than (+)-PQ. A recent pharmacokinetic study in human [15] showed that CPQ appeared readily in plasma after the administration of racemic primaquine and at peak, its concentration was 15 times higher from (−)-PQ than from (+)-PQ. In fact, in some human subjects, the observed CPQ was almost exclusively of the minus enantiomer [15]. Thus, reactions on the amino side-chain appeared to be the major clearance pathway of (−)-PQ.

Only two ring-hydroxylated PQ metabolites attributable to CYP pathway were present in substantial amounts. They were identified by accurate mass as quinone-imine metabolites of hydroxylated PQ and CPQ (*m/z* 274 and 289 respectively). Traces of other hydroxylated metabolites were observed, including the 2-OH and 4-OH-PQ, and three other quinone imines (*m/z* 274). The position of the carbonyl group of these metabolites could not be determined by mass analysis. Stability of the keto-imines however, suggests that the keto group is unlikely to be present at C-5 of the quinoline ring, as for this metabolite, a rapid degradation with demethylation of the 6-methoxy is observed.

In previous studies with recombinant human CYP2D6, substantial depletions of PQ enantiomers (50 and 30 % for (+)- and (−)-PQ, respectively) were observed during a 2 h incubation period, and CYP2D6 preferentially converted (+)-PQ to its PQ-5,6-orthoquinone and 2-hydroxyprimaquine [12]. Meanwhile, in the current study, only trace amounts of primary ring oxidative products were detected with hepatocyte incubation, all of which were exclusively generated from the (+)-PQ. The low quantities of the primary ring hydroxylation products, especially those derived from the 5-OH PQ, which has been deemed an important active metabolite [25] could possibly be due to rapid degradation or conjugation with other products. Further studies are required to resolve this issue.

The formation of the phase II conjugation reaction metabolite, identified as an *N*-carbamoyl glucuronide of PQ accounted for most of the (+)-PQ consumption. Identity of *N*-carbamoyl glucuronide of PQ was confirmed by comparison of its HPLC retention time and MS/MS fragmentation properties with the synthetic standard. It accumulated linearly over time and was the major product after prolonged incubation of PQ with human hepatocytes. The formation of this carbamoyl glucuronide of PQ as a putative metabolite in the incubation mixture of racemic PQ with pooled human hepatocytes was previously reported [26]. In the current studies, this appears to be the quantitatively predominant metabolite of (+)-PQ in the human hepatocytes, and we have also observed it in plasma and urine of human subjects receiving primaquine (manuscript in preparation). Such *N*-carbamoyl glucuronides are unusual, but have been reported as minor products for other drugs containing primary and secondary amines [27, 28]. Incubation of these drugs in a high CO_2_ environment with UDP-glucuronyltransferases can readily yield the carbamoyl glucuronides. It is, however, somewhat surprising that in the hepatocytes, this product is enantioselectively formed.

Another unexpected metabolite observed was an apparent glucose conjugate of PQ. The biphasic kinetics of formation of PQ-glucose conjugate suggests that formation of this metabolite may also depend on other metabolic reactions in the hepatocytes. It would be interesting to further investigate the pathway(s) involved in formation of this metabolite. The earlier small peak of formation of PQ-glucose conjugate may not be enzyme-dependent, since formation of this conjugate could be observed even in cell-free control incubations.

As observed against time of incubation, metabolite profiles showed different shapes, suggesting variation in kinetics and disposition. The interplay of factors including multiple enzyme involvement, dependence of one metabolite on the formation of the other, possible interactions among the metabolites might be responsible for the varying kinetics of formation of the different metabolites.

## Conclusion

The enantiomers of PQ showed significant variation in in vitro pharmacokinetics as well as metabolite generation in human hepatocytes. The in vitro clearance of (−)-PQ is three times faster than that of (+)-PQ in hepatocytes. Trace amounts of ring oxidation products were observed with (+)-PQ while (−)-PQ was preferentially metabolized by oxidative deamination of the terminal amine on the *N-*8 alkyl amino side-chain. Overall, the ring oxidation products appeared to be formed in limited amounts. A phase II conjugation reaction metabolite *N-*carbamoyl glucuronide of PQ was identified as a major metabolite, which was exclusively formed from (+)-PQ. Another PQ conjugate formed by apparent *N-*glucosylation was preferentially formed with (+)-PQ. These findings enhance the understanding of the pathways of PQ metabolism, and further inform the enantioselectivity of its metabolic profiles.
